# Association of coffee consumption with the prevalence of hearing loss in US adults, NHANES 2003–2006

**DOI:** 10.1017/S1368980023001271

**Published:** 2023-11

**Authors:** Shan Wu, Shiheng Zhu, Fengxin Mo, Xiaojing Yuan, Qiutong Zheng, Yan Bai, Wenhan Yang, Qingsong Chen

**Affiliations:** 1 Guangdong Provincial Engineering Research Center of Public Health Detection and Assessment, School of public health, Guangdong Pharmaceutical University, Guangzhou 510310, China; 2 NMPA Key Laboratory for Technology Research and Evaluation of Pharmacovigilance, Guangdong Pharmaceutical University, Guangzhou, China

**Keywords:** Hearing loss, Coffee, NHANES, Adult, Risk assessment

## Abstract

**Objective:**

This study aims to explore the association between coffee consumption and the prevalence of hearing loss in American adults based on a national population-based survey.

**Design:**

Cross-sectional analysis of reported audiometric status and coffee intake from the 2003–2006 National Health and Nutrition Examination Survey (NHANES). Multivariate logistic regression, forest plots and restricted cubic spline (RCS) analyses were used to explore the associations and dose–response relationships between coffee consumption frequency and hearing loss.

**Setting:**

The USA.

**Participant:**

This study included 1894 individuals aged ≥ 20 from the 2003–2006 NHANES.

**Results:**

In this study, the prevalence of speech-frequency hearing loss (SFHL) and high-frequency hearing loss (HFHL) among the participants was 35·90 % and 51·54 %, respectively. Compared with those who no consumed coffee, non-Hispanic White who consumed ≥ 4 cups/d had higher prevalence of SFHL (OR: 1·87; 95 % CI: 1·003. 3·47). And a positive trend of coffee consumption frequency with the prevalence of HFHL was found (*P*_trend_ = 0·001). This association of HFHL was similar for participants aged 20–64 (*P*_trend_ = 0·001), non-Hispanic White (*P*_trend_ = 0·002), non-noise exposure participants (*P*_trend_ = 0·03) and noise-exposed participants (*P*_trend_ = 0·003). The forest plots analysis found that the association between 1 cup-increment of daily coffee consumption and the prevalence of HFHL was statistically significant in males. RCS model supported a positive linear association of coffee consumption with SFHL (*P* for overall association = 0·02, *P* for nonlinearity = 0·48) and a positive non-linear association of coffee consumption with HFHL (*P* for overall association = 0·001, *P* for nonlinearity = 0·001).

**Conclusion:**

Our findings suggested that coffee consumption was associated with higher prevalence of hearing loss. Further cohort studies in larger population are needed to investigate these findings.

Hearing loss is a major global public health problem. Approximately, one-fifth of the global population currently suffer from hearing loss^(1)^. In America, nearly a quarter of people aged 12 years and over have hearing loss, including mild and unilateral hearing loss^(2)^. The WHO has estimated that hearing loss will be one of the main causes of disease burden globally by 2030^(3)^. Hearing loss may be associated with a variety of diseases. Evidence from epidemiological studies showed that hearing loss is related to the increased risks of depressive symptoms, falls, total mortality and heart disease mortality^(4–6)^. The traditional risk factors, including age, noise exposure, family history of hearing loss, exposure to ototoxic medications, smoking and diabetes, could only partially explain the causes of hearing loss^(7)^. Recently, accumulating studies have suggested that diet is related to hearing loss, and this effect can be attributed to specific dietary patterns or some special bioactive compounds, such as PUFA, vitamin A, isoflavone, riboflavin, niacin and retinol^(8–11)^.

Coffee is one of the most popular beverages in the world. Coffee and its compounds, such as caffeine, trigonelline, polyphenols and chlorogenic acid, have various impacts on human health^(12)^. Coffee trigonelline has anti-microbial, anti-carcinogenic and anti-hyperglycemic effects^(13)^. Coffee chlorogenic acid has some anti-cancer effects^(14)^. Caffeine has protective effect on neurodegenerative disease due to its strong antioxidant, anti-inflammatory and adenosine receptor antagonist properties^(15)^. It may also be related to increased risk of fractures and decreased sleep quality^(16,17)^. Additionally, polyphenols, such as caffeic acid and caffeic acid phenethyl ester, have antioxidant effects and prevent hearing loss^(18,19)^. However, caffeine are often considered a cause of tinnitus^(20)^. Large population-based studies on the association between coffee consumption and hearing loss are limited.

Currently, study about the effect of coffee consumption on hearing loss is scarce, and the results remain controversial. Two previous population-based studies have suggested a negative association between coffee consumption frequency and hearing loss^(40,41)^. Contrary, previous animals studies have shown that caffeine in coffee can interfere with hearing recovery after acoustic overstimulation events^(31,32)^. Moreover, a previous study based on the National Health and Nutrition Examination Survey (NHANES) has reported no significant association between urinary caffeine metabolites and hearing thresholds^(39)^. Therefore, in this study, we have investigated the association of coffee consumption with the hearing loss in adults from America according to the NHANES database.

## Methods

### Study design and participants

**Fig. 1:**
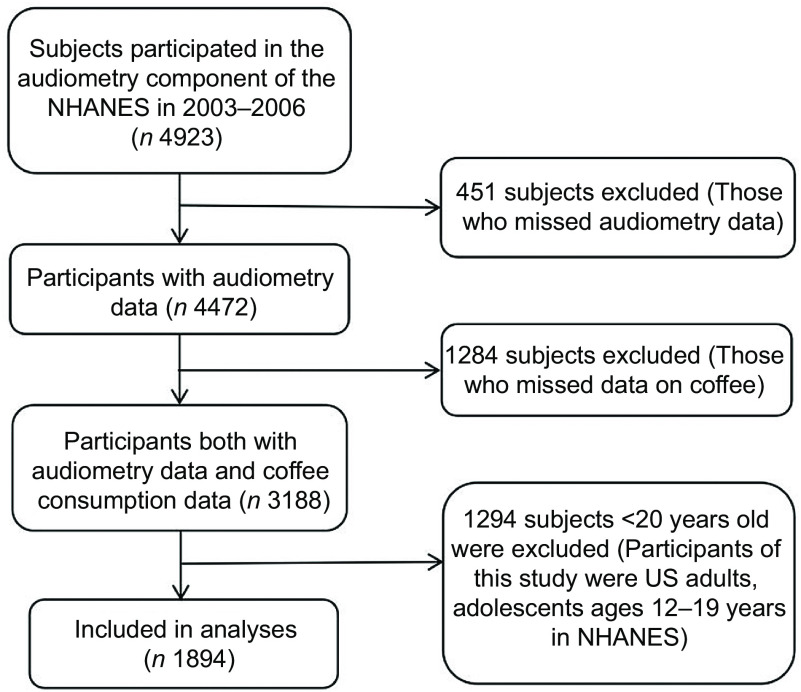
Flow chart of the selection process

The data in this study were from the NHANES database, which is a national survey administered by the National Center for Health Statistics (NCHS). This survey investigated about 10 000 representative samples of the general American population per cycle using a complex, multistage, probability sampling design. The data from the 2003–2006 were used in our study, because the information on coffee consumption and audiometry data of adult participants were collected in the same period. In the 2003–2006 NHANES, a total of 4923 subjects participated in the audiometry component. Among 1735 subjects were excluded because they lacked audiometry test data (*n* 451) and coffee consumption data (*n* 1284). Of the remaining 3188 participants, additional 1294 were eliminated because they aged < 20 years (adolescents aged 12–19 years in NHANES). Finally, 1894 subjects were recruited for analyses in this study (Fig. 1).

### Assessment of coffee consumption

Coffee consumption frequency was calculated by the FFQ, which was developed by the National Institutes of Health, National Cancer Institute (NCI) based on the NCI Diet History Questionnaire. Participants were asked to review and fill in their coffee consumption in the past 12 months. The question on coffee consumption frequency in the questionnaire was: ‘How many cups of caffeinated or decaffeinated coffee did you drink’. The possible responses were none, ≤ 1 cup/m, 1–3 cups/m, 1 cup/w, 2–4 cups/w, 5–6 cups/w, 1 cup/d, 2–3 cups/d, 4–5 cups/d, ≥ 6 cups/d. If they select other option than ‘none’, participant need to answer the following question: ‘How often was you drank the decaffeinated coffee’. The possible responses were ‘almost never or never, about 1/4 of the time, about 1/2 of the time, about 3/4 of the time, almost always or always’. In this survey, a cup of coffee was 8oz according to the Measuring Guides for the Dietary Recall Interview^(26)^.

In present analysis, total coffee consumption frequency was categorised into five groups: none, ≤ 1 cup/d, 1 cup/d, 2–3 cups/d and ≥ 4 cups/d. Then coffee consumption frequency data were further converted to quantitative data (e.g. 1–3 cups/m was converted to 0·07 cups/d). The prevalence of hearing loss related to caffeinated and decaffeinated coffee consumption was also investigated.

### Audiometric measurement

All hearing measurements were conducted by a well-trained physicians in a dedicated, sound-isolating room at a mobile examination centre. The test equipment included AD226 audiometer (Interacoustics AS, Assens, Denmark), TDH-39 standard headphones (Interacoustics AS, Assens, Denmark) and EARtone 3A insert earphones (Etymotic Research, Elk Grove Village, IL). The hearing threshold for each ear was measured at frequencies of 500, 1000, 2000, 3000, 4000, 6000 and 8000 Hz across an intensity range of –10 to 120 dB using the modified Hughson-Westlake procedure and invoking the automated testing mode of the audiometer. More details of audiometric measures are displayed on NHANES website. In our study, speech-frequency hearing loss (SFHL) was defined as pure-tone average of hearing thresholds at 500, 1000, 2000 and 4000 Hz is > 25 dB in either ear, and high-frequency hearing loss (HFHL) was defined as pure-tone average of hearing thresholds at 3000, 4000 and 6000 Hz is > 25 dB in either ear^(27)^.

### Other variables

Covariates were obtained from the questionnaire survey, including general characteristics (age, gender and ethnicity), lifestyles (smoking status, and drinking status), noise exposure and history of diseases (hypertension and diabetes mellitus). Ethnicity was classified as non-Hispanic White, non-Hispanic Black and other. Smoking status was categorised as never smoker, former smoker and current smoker. Drinking status was categorised as never drinker, low to moderate drinker (drinking < 1 drink/d in women and < 2 drinks/d in men) and heavy drinker (≥ 1 drink/d in female and ≥ 2 drinks/d in male)^(28)^. BMI status was classified as BMI < 25, BMI 25–30 and BMI ≥ 30^(29)^. The question on non-occupational noise exposure was: ‘Outside of a job, have you ever been exposed to steady loud noise or music for 5 or more hours a week? This is noise so loud that you have to raise your voice to be heard’. The possible responses were ‘yes’ or ‘no’. The question on occupational noise exposure was: ‘have you ever had a job where you were exposed to loud noise for 5 or more hours a week (you had to raise your voice to be heard)’. The possible responses were ‘yes’ or ‘no’. In addition, diagnoses of diabetes mellitus and hypertension and history of ear infection were self-reported by the subjects.

### Statistical analyses

Given the design of complex, multistage, probability sampling in the NHANES, we implemented sampling weight, cluster and strata in the analysis. Continuous variables were expressed as mean and sd for the normal distribution and as median (P_25_, P_75_) for skewed distribution and analysed by Student’s *t* tests. Categorical variables were expressed as frequency (%) and analysed by *χ* ^*2*^ test or Wilcoxon rank-sum test.

Multivariate logistic regression models were applied to assess the relationships between coffee consumption frequency and the prevalence of SFHL and HFHL. The ‘None’ group were considered as the reference groups. Covariates including ethnicity, BMI, ear infection, occupational noise exposure, non-occupational noise exposure, smoking status, drinking status, hypertension and diabetes mellitus were adjusted. The trend test was conducted by taking the median of each coffee consumption frequency group as a continuous variable in these models. We also transformed coffee consumption frequency into continuous variable to explore the linear dose–response relationship. We also conducted subgroup analyses stratified by demographic characteristics (including age (20–64 years, ≥ 65 years), ethnicity (non-Hispanic White, non-Hispanic Black and other race) and sex (male and female)), noise exposure source (including Yes (at work exposure Yes and/or outside work exposure Yes), No (at work exposure No and outside work exposure No), noise exposure unknown (outside work exposure No and at work exposure data missing)) and coffee type (caffeinated coffee, decaffeinated coffee, both). Moreover, we created forest plots to estimate the OR (95 % Cl) of hearing loss related to a 1-cup/d increment for the different types of coffee separately in men and women. Furthermore, restricted cubic splines (RCS) regression model was conducted to further explore the dose–response relationship of coffee consumption with the prevalence of SFHL and HFHL in the multivariable-adjusted binary logistic regression analyses for sex, ethnicity and noise exposure status separately, with four knots of at the 5th, 35th, 65th and 95th percentiles. Adjusted factors were consistent with multivariate logistic regression model. *P* for non-linearity < 0·05 suggested a non-linear association; otherwise, a linear association was indicated. RCS analyses was conducted using SAS macro program %RCS_Reg^(30)^. All statistical analyses were performed using SAS software (version 9.4; SAS Institute).

## Results

### Characteristics of study population

As shown in Table 1, a total of 1894 participants were included. Compared to non-coffee drinkers, those who with more consumption were older and were more likely to be non-Hispanic White and current smokers and heavy drinkers. Also, they had a lower proportion of BMI ≥ 30 and were more likely to have a diagnosis of hypertension and hearing loss; by contrast, they were more exposed to occupational noise exposure. No statistical differences between different coffee consumption frequency groups in sex, BMI, ear infection, non-occupational noise exposure and diabetes mellitus were observed (all *P* > 0·05).

**Table 1:** Characteristics of study subjects by coffee consumption

	Frequency of coffee consumption												
	Total		None		< 1cup/d		1 cup/d		2–3 cups/d		≥ 4 cups/d		
Symptoms by group	*n*	%	*n*	%	*n*	%	*n*	%	*n*	%	*n*	%	*P* value
Participants, n	1894		446		474		333		504		137		
Age (year)													
Mean	49·66		41·35		44·62		55·95		56·55		56·05		0·001***
95 % CI	47·67, 51·64		39·12, 43·57		41·44, 47·81		53·08, 58·82		53·77, 59·32		52·50, 59·60		
Sex													0·87
Male	897	47·36	195	43·72	201	42·41	158	47·45	268	53·17	75	54·74	
Female	997	52·64	251	56·28	273	57·59	175	52·55	236	46·83	62	45·26	
BMI													0·36
< 25	639	33·74	165	37·00	148	31·22	109	32·73	166	32·94	51	37·23	
25–30	647	34·16	128	28·70	164	34·60	122	36·64	181	35·91	52	37·96	
≥ 30	608	32·10	153	34·30	162	34·18	102	30·63	157	31·15	34	24·82	
Ethnicity													0·001***
Non-Hispanic White	1139	60·14	251	56·28	217	45·78	189	56·76	372	73·81	110	80·29	
Non-Hispanic Black	326	17·21	110	24·66	122	25·74	40	12·01	43	8·53	11	8·03	
Other race	429	22·65	85	19·06	135	28·48	104	31·23	89	17·66	16	11·68	
Loud noise exposure													
At work, n(%)	518	37·62	108	33·03	103	31·50	101	37·97	163	44·54	43	47·25	0·04*
Missing	517		119		147		67		138		46		
Outside work	427	22·57	111	24·89	103	21·73	61	18·32	116	23·06	36	26·47	0·18
SFHL													0·001**
Yes	680	35·90	104	23·32	126	26·58	150	45·05	234	46·43	66	48·18	
No	1214	64·10	342	76·68	348	73·42	183	54·95	270	53·57	71	51·82	
HFHL													0·001**
Yes	970	51·54	145	32·73	192	40·51	211	63·94	330	66·27	92	67·15	
No	912	48·46	298	67·27	282	59·49	119	36·06	168	33·73	45	32·85	
Ear infection	83	4·54	19	4·48	22	4·88	11	3·41	20	4·03	11	8·27	0·55
Smoking status													0·001***
Never smoker	927	49·00	277	62·11	282	59·62	171	51·35	176	34·92	21	15·44	
Former smoker	586	30·97	94	21·08	112	23·68	123	36·94	200	39·68	57	41·91	
Current smoker	379	20·03	75	16·82	79	16·70	39	11·71	128	25·40	58	42·65	
Drinking status													0·01**
Never drinker	591	32·31	186	43·66	147	32·59	105	32·61	115	23·23	38	28·15	
Low to moderate drinker	1081	59·10	216	50·70	272	60·31	190	59·01	318	64·24	85	62·96	
Heavy drinker	157	8·58	24	5·63	32	7·10	27	8·39	62	12·53	12	8·89	
History of diseases													
Hypertension	728	38·58	136	30·63	165	34·96	154	46·39	223	44·42	50	36·50	0·001***
Diabetes mellitus	212	11·19	35	7·85	44	9·28	44	13·21	69	13·69	20	14·60	0·10

SFHL, speech-frequency hearing loss; HFHL, high-frequency hearing loss.

*P* values from *χ*^*2*^ test or Wilcoxon rank-sum test (categorical categories) and Student’s *t* tests (continuous covariates).

* *P* < 0·05.

** *P* < 0·01.

*** *P* < 0·001.

### Association between coffee consumption and hearing loss risk

As shown in Table 2, there was no correlation between coffee consumption and the prevalence of SFHL and HFHL in all groups in the full-adjusted model. A positive trend and association of coffee consumption frequency with the prevalence of SFHL was found in crude model (*P*_trend_ = 0·001), but this trend has insignificant after full adjustments (*P*_trend_ = 0·25). Besides, a positive trend and association of coffee consumption frequency with the prevalence of HFHL in crude model was found (*P*_trend_ < 0·05), while only the trend remained after full adjustments (*P*_trend_ < 0·05)

**Table 2:** OR and 95 % CI of coffee consumption for hearing loss

	Frequency of coffee consumption									
		< 1 cup/d		1 cup/d		2–3 cups/d		≥ 4 cups/d		
	None	OR	95 % CI	OR	95 % CI	OR	95 % CI	OR	95 % CI	*P* trend
SFHL										
Total (*n* 1894)										
Cases/*n*	104/446	126/474		150/333		234/504		66/137		
Crude model	Ref	1·28	0·80, 2·04	2·57	1·52, 4·36‡	2·55	1·57, 4·15‡	2·82	1·65, 4·81‡	0·001*
Model 1†	Ref	1·12	0·61, 2·04	1·46	0·83, 2·57	1·27	0·68, 2·37	1·54	0·81, 2·91	0·25
HFHL										
Total (*n* 1882)										
Cases/*n*	145/443	192/474		211/330		330/498		92/137		
Crude model	Ref	1·32	0·87, 2·02	3·26	2·03, 5·26‡	3·94	2·66, 5·86‡	3·72	2·34, 5·93‡	0·001*
Model 1†	Ref	1·18	0·63, 2·21	2·41	1·28, 4·55‡	2·61	1·51, 4·50‡	1·89	0·90, 3·99	0·001*

SFHL, speech-frequency hearing loss; HFHL, high-frequency hearing loss.

* *P* trend < 0·05.

† Adjusted for age, sex, ethnicity, ear infection, occupational noise exposure, non-occupational noise exposure, smoking status, drinking status, hypertension, diabetes mellitus and BMI.

‡ *P* < 0·05.

### Subgroup analyses

The results of subgroup analyses were shown in Table 3, online Supplementary Table S1 and S2. In non-Hispanic White subgroup, only participants who consumed ≥ 4 cups/d had a higher prevalence of SFHL compared to non-coffee drinker group (OR: 1·87; 95 % CI: 1·003, 3·47; *P* = 0·049, Table 3). Nevertheless, no trends and association of coffee consumption frequency with the prevalence of SFHL in subgroups of age (20–64 years, ≥ 65), ethnicity (non-Hispanic Black and other race) and sex (male and female) were observed (*P*_trend_ > 0·05, Table 3). Besides, positive trends of coffee consumption frequency with the prevalence of HFHL in subgroups of age (20–64 years), ethnicity (non-Hispanic White) and sex (male and female) were observed (*P*_trend_ < 0·05, Table 3). Moreover, in noise exposure subgroup, a positive trend of coffee consumption frequency with the prevalence of SFHL in Model 1 was observed (*P*_trend_ = 0·048, online Supplementary Table S1). Similarly, the positive trend of coffee consumption frequency with the prevalence of HFHL in Model 1 was also found in subgroups of loud noise exposure (yes and no). In addition, no significant correlations were also found between coffee consumption frequency and the prevalence of SFHL and HFHL in all coffee type subgroups (online Supplementary Table S2).

**Table 3:** OR and 95 % CI of coffee consumption for hearing loss stratified by demographic characteristics

	Frequency of coffee consumption									
		< 1 cup/d		1 cup/d		2–3 cups/d		≥ 4 cups/d		
	None	OR	95 % CI	OR	95 % CI	OR	95 % CI	OR	95 % CI	*P* trend
SFHL										
Age										
Age 20–64 years (*n* 1194)										
Cases/*n*	35/348	37/337		27/174		57/259		20/76		
Crude model	Ref	1·28	0·65, 2·54	1·62	0·82, 3·20	2·07	0·98, 4·38	2·50	1·01, 6·21‡	0·03*
Model 1†	Ref	1·17	0·48, 2·83	1·29	0·63, 2·64	1·32	0·62, 2·84	1·31	0·63, 2·75	0·46
Age ≥ 65 years (*n* 700)										
Cases/*n*	69/98	89/137		123/159		177/245		46/61		
Crude model	Ref	0·85	0·42, 1·70	1·43	0·75, 2·73	0·99	0·50, 1·97	1·38	0·50, 3·78	0·58
Model 1†	Ref	0·88	0·44, 1·75	1·25	0·67, 2·33	0·94	0·48, 1·84	1·16	0·39, 3·48	0·82
Ethnicity										
Non-Hispanic White (*n* 1139)										
Cases/*n*	68/251	77/217		101/189		195/372		54/110		
Crude model	Ref	1·48	0·82, 2·67	2·60	1·35, 5·03‡	2·77	1·63, 4·73‡	2·62	1·58, 4·32‡	0·001*
Model 1†	Ref	1·22	0·58, 2·58	1·52	0·70, 3·29	1·62	0·82, 3·17	1·87	1·003, 3·47‡	0·07
Non-Hispanic Black (*n* 326)										
Cases/*n*	17/110	24/122		15/40		6/43		4/11		
Crude model	Ref	1·60	0·50, 5·11	3·05	1·02, 9·07‡	0·62	0·14, 2·76	3·51	0·95, 12·95	0·55
Model 1†	Ref	1·51	0·33, 6·89	0·48	0·09, 2·50	0·13	0·01, 1·80	0·29	0·03, 2·46	0·10
Other race (*n* 429)										
Cases/*n*	19/85	25/135		34/104		33/89		8/16		
Crude model	Ref	0·64	0·28, 1·43	2·08	0·72, 6·04	0·86	0·37, 2·00	3·01	0·53, 17·12	0·20
Model 1†	Ref	0·51	0·18, 1·47	0·80	0·27, 2·40	0·37	0·10, 1·35	0·49	0·07, 3·36	0·24
Sex										
Male (*n* 897)										
Cases/*n*	64/195	60/201		80/158		149/268		44/75		
Crude model	Ref	1·21	0·60, 2·44	2·23	1·02, 4·87‡	2·84	1·46, 5·52‡	3·30	1·41, 7·69‡	0·001*
Model 1†	Ref	1·10	0·48, 2·51	1·16	0·55, 2·46	1·39	0·64, 3·02	1·50	0·58, 3·89	0·37
Female (*n* 997)										
Cases/*n*	40/251	66/273		70/175		85/236		22/62		
Crude model	Ref	1·31	0·70, 2·45	2·94	1·56, 5·53‡	2·29	1·13, 4·64‡	2·22	1·02, 4·85‡	0·002*
Model 1†	Ref	0·87	0·37, 2·06	1·53	0·62, 3·77	0·95	0·34, 2·67	1·23	0·50, 3·02	0·72
HFHL										
Age										
Age 20–64 years (*n* 1194)										
Cases/*n*	60/348	71/337		61/174		111/258		37/77		
Crude model	Ref	1·26	0·76, 2·10	2·33	1·35, 4·02‡	3·57	2·12, 6·00‡	3·49	1·74, 6·99‡	0·001*
Model 1†	Ref	1·19	0·60, 2·36	2·30	1·12, 4·69‡	2·91	1·69, 5·03‡	1·75	0·75, 4·11	0·001*
Age ≥ 65 years (*n* 688)										
Cases/*n*	85/95	121/137		150/156		219/240		55/60		
Crude model	Ref	0·81	0·22, 2·95	3·37	0·75, 15·13	1·09	0·34, 3·49	1·74	0·35, 8·81	0·50
Model 1†	Ref	1·23	0·29, 5·22	3·98	0·83, 19·08	1·39	0·40, 4·85	2·10	0·50, 8·81	0·32
Ethnicity										
Non-Hispanic White (*n* 1127)										
Cases/*n*	93/248	105/217		134/186		267/367		77/109		
Crude model	Ref	1·53	0·97, 2·42	3·03	1·83, 5·05‡	3·94	2·51, 6·17‡	3·39	2·10, 5·47‡	0·001*
Model 1†	Ref	1·24	0·66, 2·36	2·01	1·02, 3·94‡	2·83	1·49, 5·37‡	2·29	1·06, 4·93‡	0·002*
Non-Hispanic Black (*n* 327)										
Cases/*n*	22/110	44/122		22/40		15/43		7/12		
Crude model	Ref	2·22	1·08, 4·55‡	6·02	1·99, 18·18‡	2·24	0·83, 6·06	4·52	1·10, 18·51‡	0·01*
Model 1†	Ref	4·50	1·24, 16·39‡	5·86	0·57, 60·54	1·79	0·47, 6·77	0·26	0·04, 1·69	0·66
Other race (*n* 428)										
Cases/*n*	30/85	43/135		55/104		48/88		8/16		
Crude model	Ref	0·64	0·28, 1·49	3·22	1·56, 6·65‡	2·31	0·64, 8·35	1·51	0·34, 6·60	0·03*
Model 1†	Ref	0·31	0·10, 0·90‡	2·22	0·98, 5·03	1·27	0·36, 4·49	0·07	0·01, 0·33‡	0·56
Sex										
Male (*n* 887)										
Cases/*n*	88/192	96/201		110/155		203/264		62/75		
Crude model	Ref	1·09	0·58, 2·04	2·16	0·98, 4·76	4·08	2·57, 6·47‡	4·98	2·39, 10·37‡	0·001*
Model 1†	Ref	0·84	0·38, 1·87	1·42	0·63, 3·19	2·33	1·44, 3·78‡	1·70	0·81, 3·57	0·001*
Female (*n* 995)										
Cases/*n*	57/251	96/273		101/175		127/234		30/62		
Crude model	Ref	1·59	0·89, 2·85	4·93	3·06, 7·94‡	4·31	2·30, 8·05‡	3·05	1·54, 6·03‡	0·001*
Model 1†	Ref	1·34	0·50, 3·60	3·85	1·71, 8·68‡	2·62	0·90, 7·66	1·72	0·54, 5·49	0·04*

SFHL, speech-frequency hearing loss; HFHL, high-frequency hearing loss.

* *P* trend < 0·05.

† Adjusted for age, sex, ethnicity, ear infection, occupational noise exposure, non-occupational noise exposure, smoking status, drinking status, hypertension, diabetes mellitus, and BMI.

‡ *P* < 0·05.

### The forest plot analysis of association of coffee consumption with hearing loss

**Fig. 2:**
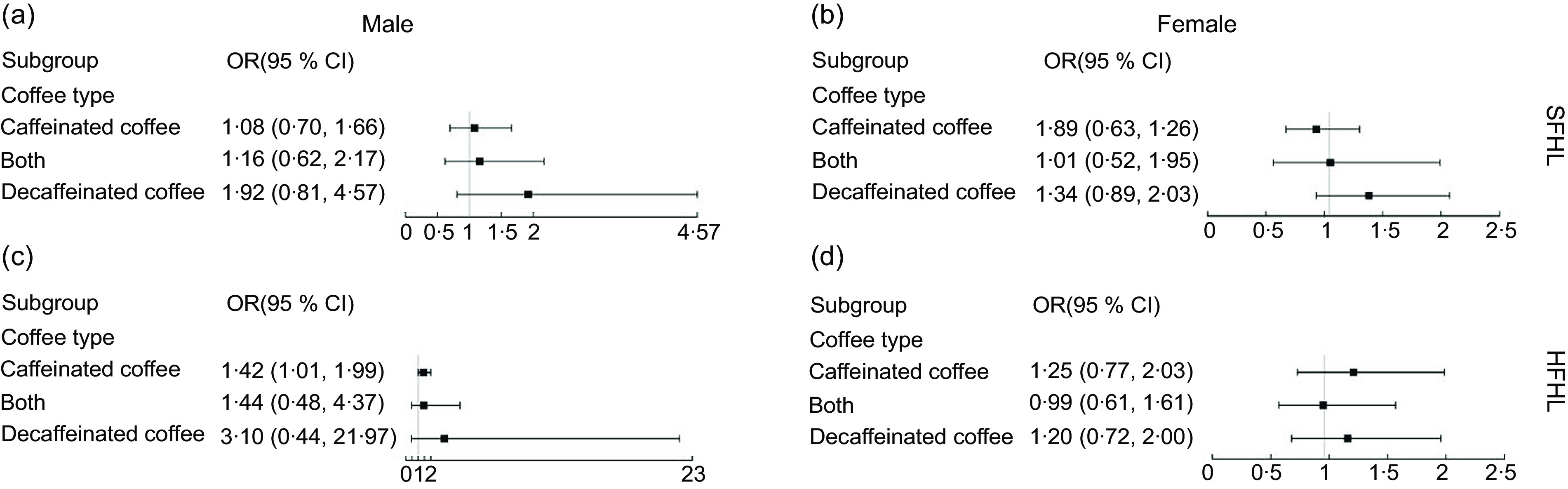
OR (95 % CI) per 1 cup-increment for the association between the different types of coffee and the prevalence of hearing loss stratified by sex. (a) and (b) speech-frequency hearing loss (SFHL). (c) and (d) High-frequency hearing loss (HFHL). Analyses are adjusted for age, ethnicity, ear infection, occupational noise exposure, non-occupational noise exposure, smoking status, drinking status, hypertension, diabetes mellitus and BMI

The relationship between coffee consumption and the prevalence of SFHL and HFHL appeared to be more pronounced in male subgroup. However, only in caffeinated coffee subgroup, the association between per 1 cup-increment of daily coffee consumption and HFHL was statistically significant in male (Fig. 2).

### Dose–response relationships between coffee consumption and the prevalence of hearing loss

**Fig. 3:**
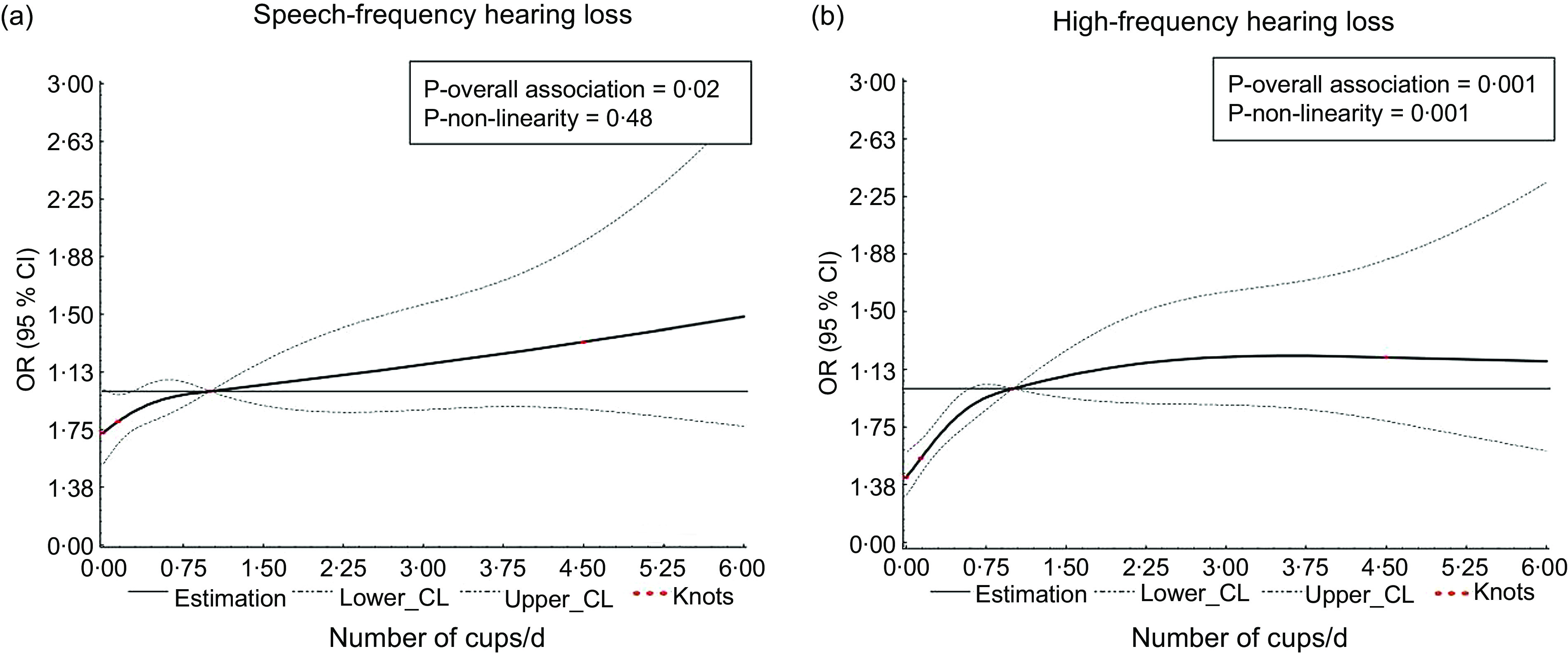
Multivariable-adjusted spline curves of relation between total coffee and the prevalence of hearing loss. (a) SFHL. (b) HFHL. Covariates were age, sex, ethnicity, ear infection, occupational noise exposure, non-occupational noise exposure, smoking status, drinking status, hypertension, diabetes mellitus and BMI. SFHL, speech-frequency hearing loss; HFHL, high-frequency hearing loss

RCS model also showed a linear positive associations of coffee consumption with the prevalence of SFHL (*P* overall association = 0·02, *P* nonlinearity = 0·48; Fig. 3 (a)), while a non-liner positive associations of coffee consumption with the prevalence of HFHL was found (*P* overall association = 0·001, *P* nonlinearity = 0·001; Fig. 3 (b)). Besides, a positive linear associations of coffee consumption with the prevalence of SFHL were found in the noise exposure subgroup (*P* overall association = 0·03, *P* nonlinearity = 0·47; online Supplementary Fig. S1 (a)). Likewise, positive linear associations of coffee consumption with the prevalence of HFHL were found in male subgroup and non-noise exposure subgroup (all *P* overall association < 0·05, *P* nonlinearity > 0·05; online Supplementary Fig. S1 (f)–(g)), while positive non-linear associations of coffee consumption with the prevalence of HFHL were found in the age 20–64 years, non-Hispanic White, female and noise exposure subgroup (all *P* overall association < 0·05, *P* nonlinearity < 0·05; online Supplementary Fig. S1).

## Discussion

In the present study, we assessed the relationship between coffee consumption and hearing loss in American adults aged ≥ 20 years from NHANES 2003–2006 and found that coffee consumption was related to the prevalence of hearing loss among US adults, especially male and participants with noise exposed. A significant trend of coffee consumption frequency with the prevalence of hearing loss was found. These results may be independent of the coffee type or the preparation method.

As a common drink, coffee has attracted increasing attention on its health effects. However, studies about the impact of coffee consumption on hearing loss were limited, and the results were controversial. In this study, we found a positive association of coffee consumption frequency with the prevalence of SFHL and HFHL in US adults. Caffeine is one of the main components of coffee. Caffeine has non-selective adenosine receptor antagonist properties, which interfers hearing recovery after acoustic overstimulation events via antagonising adenosine receptors in Corti organ, lateral wall, spiral ganglion cells and cochlear blood vessels, leading to interruption of cochlear blood reperfusion and the increased production of oxidative stress^(31–33)^. Also, caffeine increases the production and accumulation of Ca in the cochlear hair cells after noise exposure, which activate Ca-dependent isomers and cleave Ca-dependent neurons by phospholipase A2 that lead to the apoptosis of cochlear hair cells^(23,34)^. Besides, caffeine exacerbates the noise-induced hypoperfusion and ischemia in the cochlea by promoting the reduction of cerebral blood flow and arteriole diameter^(24,35)^. Caffeine also exacerbates the physiological increase of corticosterone by altering the hypothalamic–pituitary–adrenocortical axis, thus causing an acute response to noise^(36,37)^. In addition, caffeine caused autophagy and apoptosis in the cochlear hair cells through SGK1/HIF-1*α* pathway^(38)^. However, a previous study based on the NHANES showed that urinary caffeine metabolites were not associated with the changes of hearing thresholds in US adults^(39)^. Contrary to our findings, two previous population-based studies have shown a negative association between coffee consumption frequency and hearing loss^(40,41)^. The inconsistent results can be mainly attributed to differences in the study population, the sample size, the definition of hearing loss, the measurement of hearing thresholds, the inclusion and exclusion criteria, and the covariates included in statistical model^(42)^.

Of note, our study showed a positive trend and association of coffee consumption frequency with the prevalence of SFHL and HFHL in non-Hispanic Whites, and participants who consumed ≥ 4 cups/d coffee had a 1·87-fold higher prevalence of SFHL than non-coffee drinkers. Similar to our finding, another study based on the NHANES database also showed that the odds of hearing loss are substantially higher in non-Hispanic White Americans than in other ethnic individuals (OR: 2·3; 95 % CI: 1·3, 3·9)^(25)^. Skin pigmentation, as a marker of melanocytic functioning, may mediate the close relationship of race/ethnicity and hearing loss^(21)^. Genes also play an important role in the occurrence and development of hearing loss. It has been reported that the prevalence of hearing loss caused by pathogenic autosomal recessive non-syndromic (ARNS) HI genes varies from race to race, and African Americans/African Americans receive the least impact^(22)^. In addition, we also found that even after adjusting for covariates related to hearing loss, there was still a sex difference in the relationship between coffee and hearing loss, the association between per 1 cup-increment of daily coffee consumption and HFHL was statistically significant in men, but no significant association was found among women, which was similar to a previous study^(2)^. In this study, the proportion of male coffee consumers is higher than that of female (78·26 % *v*. 74·82 %). Coffee drinking is widespread in the USA, and men consumed more coffee^(43,44)^. Previous studies have shown that men may have a higher hearing threshold^(2)^. It may be due to the great differences between men and women in brain biochemistry, physiology, structure and function. In physiological structure, the length of the cochlea in men was longer than that in women, which could affect the auditory brainstem responses. Besides, oestrogen also plays a protective role in the cochlear function^(45)^. Oestrogen may play an important role in modulating the pathophysiological mechanisms in the hearing system, and it could enhance the expression of antioxidant superoxide dismutase and decrease apoptosis by upregulating Bcl-2/Bcl-xL and inhibiting the JNK pathway, and it also could inhibit glutamate excitotoxicity to regulate cochlear homoeostasis^(46)^. Thus, in women, the effect of nutrition on auditory function may not be as relevant as in men. Besides, we found a positive trend and relationship between coffee consumption frequency with the prevalence of HFHL in non-noise exposure subgroup, suggesting that caffeine may have a potential effect on hearing. A previous study reported that caffeine significantly suppressed the compound action potential of the auditory nerve after infusing caffeine into the perilymph compartment^(47)^. Noteworthy, we found a positive trend and relationship of coffee consumption frequency with the prevalence of SFHL and HFHL in the noise-exposed participants. Noise-induced hearing loss mainly refers to the apoptosis of cochlear hair cells caused by noise through mechanical damage and metabolic damage (such as oxidative stress damage, Ca^2+^ overload, and immune and inflammatory damage)^(48)^. The interaction between coffee and noise may aggravate the apoptosis of cochlear hair cells. Given the biological plausibility, it will be very meaningful to conduct further studies to establish the potential role of coffee consumption combined with noise exposure on hearing loss.

This study has several advantages. First, the data are from NHANES, which have been implemented in the USA for a long time, and the survey implementation process is rigorous and mature, so the results of this study are relatively reliable. Second, given that the survey design was complex, multistage, probability sampling in the NHANES, we conducted sampling weight, cluster and strata to solve the deviation of variance estimation caused by such clustering data in the statistical analyses. Besides, we explored the association between the prevalence of SFHL and HFHL with coffee consumption frequency. This study also has several limitations. First, since only two circles (2003–2006) in the NHANES have collected the information on coffee consumption frequency and audiometry test of American adults, the sample size was relatively small; thus, the extrapolation of this result to the general population should be more cautious. Secondly, coffee consumption is obtained through questionnaires, which may have participants’ recall bias, and it is difficult to accurately obtain information about coffee consumption, such as individual coffee intake, type of coffee consumption and preparation process. Therefore, the error of coffee consumption between measured exposure and actual exposure cannot be completely eliminated^(49)^. Finally, this was an observation study, so causality between coffee consumption and hearing loss could not be shown.

### Conclusions

This finding suggested that the positive trend and association of coffee consumption frequency with the prevalence of hearing loss in US adults. And this association was found in non-Hispanic Whites, men, aged 20–64 participants and noise-exposed individuals. This association may be independent of the coffee type or the preparation method. Further cohort studies in larger population are needed to validate these findings, and the underlying mechanism also remains to be elucidated.

## Supplementary material

For supplementary material accompanying this paper visit https://doi.org/10.1017/S1368980023001271. click here to view supplementary material
