# Supplementary material for: Association of coffee consumption with the prevalence of hearing loss in US adults, NHANES 2003–2006
Source: Public Health Nutr. 2023 Jul 24;26(11):2322–32. doi: 10.1017/S1368980023001271 (PMC10641622; doi:10.1017/S1368980023001271)
Supplement: Supplementary file 1 [file S1368980023001271sup001.docx]

Association of coffee consumption with the prevalence of hearing loss in US adults, NHANES 2003-2006

# Supplementary Figures and Tables

## Supplementary Figures

**Supplementary Figure. S1**


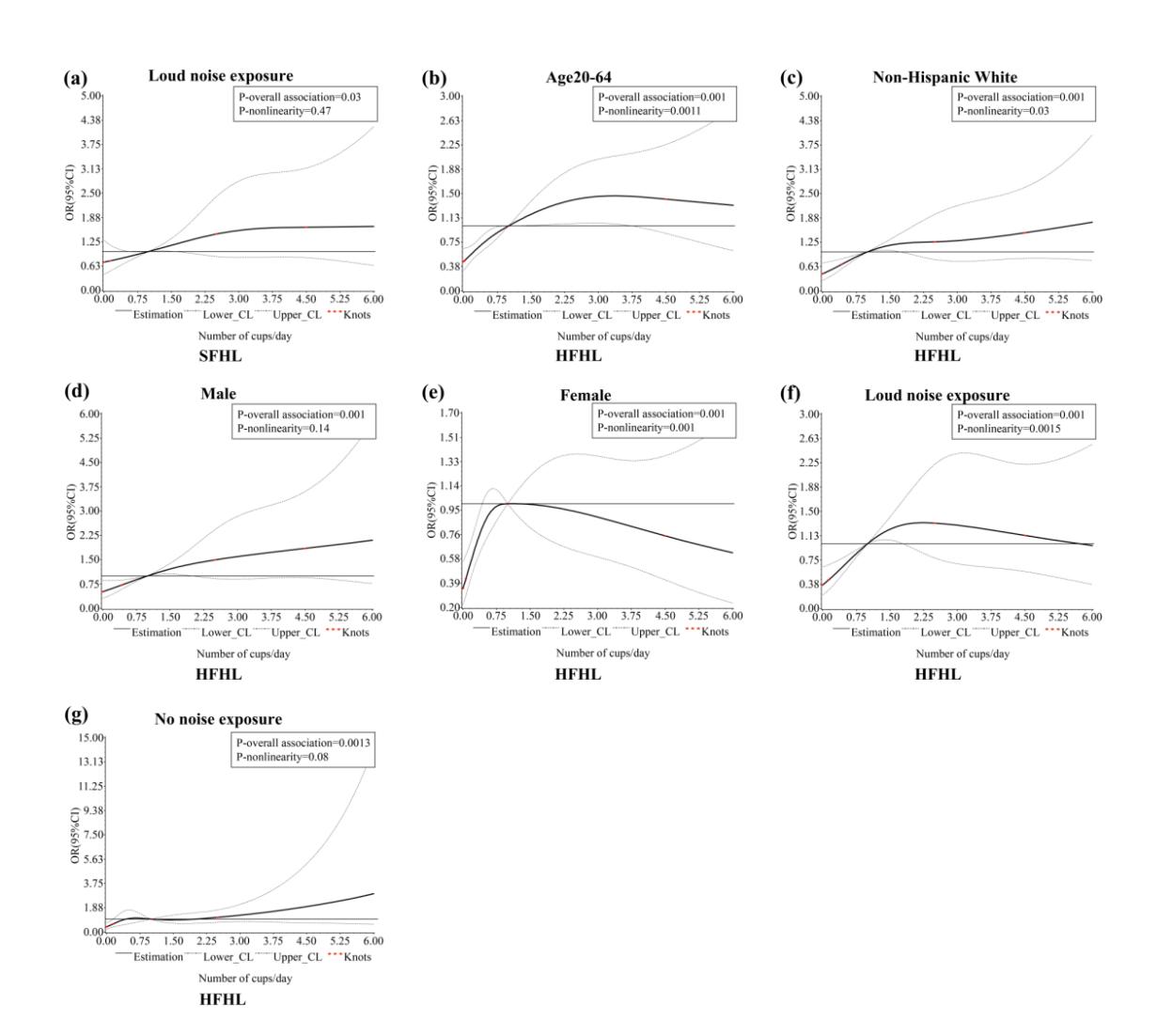


**Supplementary Figure. S1** Multivariable adjusted spline curves of relation between total coffee and the risk of hearing loss in age, sex, ethnicity and noise exposure status. (a)SFHL. (b)-(g)HFHL. Covariates were age, sex, ethnicity, ear infection, occupational noise exposure, non-occupational noise exposure, smoking status, drinking status, hypertension, diabetes mellitus, BMI. OR = odds ratios.

## Supplementary Tables

**Supplementary Table S1** Odds ratios and 95% confidence intervals of coffee consumption for hearing loss stratified by noise exposure.

|  | Frequency of Coffee Consumption | | | | | |
| --- | --- | --- | --- | --- | --- | --- |
|  | None | < 1cup/d | 1cup/d | 2-3cup/d | ≥ 4cup/d | *P* trend |
| **SFHL** |  |  |  |  |  |  |
| **Loud noise exposure** |  |  |  |  |  |  |
| **Yes (n = 774)** |  |  |  |  |  |  |
| Cases/N | 46/180 | 55/173 | 61/129 | 121/224 | 35/68 |  |
| Crude model | Ref | 1.80 (0.93, 3.46) | 2.75 (1.03, 7.36)^b^ | 3.51 (1.98, 6.23)^b^ | 3.33 (1.63, 6.81)^b^ | 0.001^*^ |
| Model 1^a^ | Ref | 1.35 (0.55, 3.31) | 1.14 (0.48, 2.75) | 1.97 (0.94, 4.13) | 2.27 (0.99, 5.20) | 0.048^*^ |
| **No (n = 727)** |  |  |  |  |  |  |
| Cases/N | 41/178 | 52/187 | 70/150 | 71/176 | 20/36 |  |
| Crude model | Ref | 1.48 (0.86, 2.54) | 2.88 (1.63, 5.09)^b^ | 2.56 (1.45, 4.51)^b^ | 3.77 (1.46, 9.76)^b^ | 0.001^*^ |
| Model 1^a^ | Ref | 1.49 (0.61, 3.65) | 1.47 (0.64, 3.39) | 0.92 (0.33, 2.55) | 1.95 (0.55, 6.94) | 0.95 |
| **Noise exposure unknown (n = 391)** |  |  |  |  |  |  |
| Cases/N | 17/88 | 19/114 | 19/54 | 42/103 | 10/32 |  |
| Crude model | Ref | 0.44 (0.20, 1.01) | 2.16 (0.38, 12.43) | 1.22 (0.31, 4.71) | 1.28 (0.62, 2.66) | 0.27 |
| Model 1^a^ | Ref | 0.39 (0.14, 1.05) | 1.37 (0.24, 7.93) | 0.57 (0.11, 3.01) | 0.46 (0.20, 1.05) | 0.46 |
| **HFHL** |  |  |  |  |  |  |
| **Loud noise exposure** |  |  |  |  |  |  |
| **Yes (n = 768)** |  |  |  |  |  |  |
| Cases/N | 61/178 | 78/173 | 84/127 | 160/222 | 48/68 |  |
| Crude model | Ref | 1.75 (0.93, 3.27) | 4.16 (1.81, 9.61)^b^ | 4.74 (2.45, 9.17)^b^ | 4.32 (2.26, 8.24)^b^ | 0.001^*^ |
| Model 1^a^ | Ref | 1.18 (0.52, 2.65) | 2.72 (0.998, 7.40) | 3.79 (1.72, 8.37)^b^ | 2.07 (0.81, 5.27) | 0.003^*^ |
| **No (n = 724)** |  |  |  |  |  |  |
| Cases/N | 55/177 | 77/187 | 97/149 | 111/175 | 28/36 |  |
| Crude model | Ref | 1.41 (0.77, 2.57) | 3.47 (1.89, 6.38)^b^ | 4.88 (2.68, 8.86)^b^ | 9.19 (2.98, 28.31)^b^ | 0.001^*^ |
| Model 1^a^ | Ref | 1.50 (0.59, 3.83) | 2.86 (1.09, 7.51)^b^ | 3.28 (0.77, 13.90) | 8.59 (1.96, 37.72)^b^ | 0.03^*^ |
| **Noise exposure unknown (n = 389)** |  |  |  |  |  |  |
| Cases/N | 29/88 | 37/114 | 30/54 | 59/101 | 15/32 |  |
| Crude model | Ref | 0.67 (0.28, 1.58) | 2.37 (0.64, 8.84) | 1.80 (0.85, 3.80) | 1.08 (0.46, 2.56) | 0.07 |
| Model 1^a^ | Ref | 0.59 (0.17, 2.00) | 2.50 (0.74, 8.37) | 0.93 (0.28, 3.10) | 0.32 (0.07, 1.48) | 0.59 |

^a^ Adjusted for age, sex, ethnicity, ear infection, smoking status, drinking status, hypertension, diabetes mellitus, BMI.

^b^ *P* < 0.05.

^*^*P* trend < 0.05.

**Supplementary Table S2** Odds ratios and 95% confidence intervals of coffee consumption for hearing loss stratified by coffee type.

|  | Frequency of Coffee Consumption | | | | |
| --- | --- | --- | --- | --- | --- |
|  | < 1cup/d | 1cup/d | 2-3cup/d | ≥ 4cup/d | *P* trend |
| **SFHL** |  |  |  |  |  |
| Both (n = 265) |  |  |  |  |  |
|  |  |  |  |  |  |
| Cases/N | 24/93 | 20/56 | 34/90 | 11/26 |  |
| Crude model | Ref | 2.01 (0.74, 5.50) | 3.07 (1.36, 6.95)^b^ | 1.90 (0.65, 5.57) | 0.03^*^ |
| Model 1^a^ | Ref | 1.23 (0.35, 4.39) | 1.86 (0.95, 3.64) | 0.65 (0.15, 2.86) | 0.79 |
| Caffeinated coffee (n = 850) |  |  |  |  |  |
| Cases/N | 43/230 | 87/202 | 150/323 | 43/95 |  |
| Crude model | Ref | 2.16 (1.12, 4.15)^b^ | 2.09 (1.13, 3.85)^b^ | 2.48 (1.04, 5.92)^b^ | 0.02^*^ |
| Model 1^a^ | Ref | 1.05 (0.42, 2.66) | 0.81 (0.34, 1.93) | 1.02 (0.32, 3.27) | 0.78 |
| Decaffeinated coffee (n = 298) |  |  |  |  |  |
| Cases/N | 55/129 | 39/70 | 48/85 | 10/14 |  |
| Crude model | Ref | 1.66 (0.87, 3.19) | 1.68 (0.72, 3.94) | 5.31 (1.83, 15.41)^b^ | 0.03^*^ |
| Model 1^a^ | Ref | 2.79 (0.89, 8.73) | 1.60 (0.47, 5.42) | 13.14 (1.49, 115.61)^b^ | 0.14 |
| **HFHL** |  |  |  |  |  |
| Both (n = 264) |  |  |  |  |  |
| Cases/N | 32/93 | 36/56 | 56/90 | 16/25 |  |
| Crude model | Ref | 7.22 (2.43, 21.48)^b^ | 4.64 (1.82, 11.82)^b^ | 2.45 (0.80, 7.47) | 0.02^*^ |
| Model 1^a^ | Ref | 7.33 (2.14, 25.07)^b^ | 2.17 (0.77, 6.07) | 0.81 (0.16, 4.05) | 0.71 |
| Caffeinated coffee (n = 844) |  |  |  |  |  |
| Cases/N | 74/230 | 120/201 | 205/317 | 63/96 |  |
| Crude model | Ref | 2.17 (1.21, 3.90)^b^ | 3.01 (1.81, 5.02)^b^ | 3.48 (1.86, 6.51)^b^ | 0.001^*^ |
| Model 1^a^ | Ref | 1.40 (0.62, 3.17) | 2.06 (1.08, 3.93)^b^ | 1.70 (0.53, 5.46) | 0.09 |
| Decaffeinated coffee (n = 297) |  |  |  |  |  |
| Cases/N | 79/129 | 51/68 | 66/85 | 11/14 |  |
| Crude model | Ref | 1.66 (0.77, 3.59) | 3.20 (1.23, 8.34)^b^ | 4.67 (1.17, 18.69)^b^ | 0.01^*^ |
| Model 1^a^ | Ref | 2.34 (0.92, 5.98) | 1.91 (0.68, 5.33) | 3.85 (0.62, 24.01) | 0.12 |

^a^ Adjusted for age, sex, ethnicity, ear infection, occupational noise exposure, non-occupational noise exposure, smoking status, drinking status, hypertension, diabetes mellitus, BMI.

^b^ *P* < 0.05.

^*^*P* trend < 0.05.
